# Rare case of bilateral anterolateral and symmetrical bowing of tibia successfully treated with a distal tibial opening wedge osteotomy

**DOI:** 10.1093/jscr/rjz224

**Published:** 2019-12-13

**Authors:** Margarida Miranda, Carolina Afonso, Carla Martins, João Carvalho, Armando Campos

**Affiliations:** 1 Department of Orthopedics, Unidade Local de Saúde do Alto Minho, Viana do Castelo, Portugal; 2 Department of Orthopedics, Unidade Local de Saúde do Nordeste, Bragança, Portugal; 3 Department of Orthopedics, Centro Hospitalar Tondela Viseu, Viseu, Portugal; 4 Department of Orthopedics, Centro Hospitalar de Tâmega e Sousa, Penafiel, Portugal; 5 Department of Orthopedics, Centro Hospitalar do Porto, Porto, Portugal

## Abstract

The anterolateral bowing of the tibia is closely associated with the development of its pseudarthrosis. Roughly, all deformities are unilateral so the shortening and angulation are easy to identify. We present a 6-year-old boy with an exuberant bilateral anterolateral bowing of tíbia. He has short stature, disturbed gait and callosity at the lateral border of the foot. Deformity was successfully treated by opening wedge tibia osteotomy and filled the remaining gap with structural fibular graft. It was fixed with two crossed K-wires and cast immobilization for 6 weeks. We decided to correct it before skeletal maturity due to the significant disturbance of the gait and esthetic impairment. It was obtained a satisfactory morphological and functional result with a simple and fast technique.

## INTRODUCTION

Angular deformities of the lower limbs are common during childhood. In most cases, this represents a variation in the normal growth pattern and is an entirely benign condition. The presence of symmetrical deformities and absence of symptoms indicate a benign condition. In contrast, deformities which are asymmetrical and associated with pain, joint stiffness, systemic disorders or syndromes may indicate a serious underlying cause and require treatment [1].

Congenital bowing leg comprises two diseases with different evolution and prognosis: anterolateral and posteromedial bowing according to the main axis of tibial angulation.

The posteromedial angular deformities of the tibia are associated with the calcaneus-valgus foot [2] and tend to improve with growth.

The anterolateral bowing of the tibia is closely associated with the development of its pseudarthrosis, after spontaneous fracture of the tibia. Pseudarthrosis in most cases of bowing of the tibia is not present at birth. Therefore, the term ‘pseudarthrosis’ might be somewhat inaccurate, because not all anterolateral deformities progress to nonunion, and dysplasia would be the preferred term [2,3,4].

Management of anterolateral bowing of the tibia and subsequent pseudarthrosis are considered as a continuum.

Although the etiology is unknown [5], the fibrous soft tissue found in the pseudarthosis and the abnormal periosteum are certainly a key to the pathology, possibly due to decreased osteogenic capacities and impaired local vascularization [6].

The relationship of anterolateral bowing and neurofibromatosis has been known since 1937 [3] but the exact pathogenesis still remains nuclear.

Although only 5% of the patients with have type 1 neurofibromatosis are diagnosed with anterolateral bowing and pseudarthrosis, up to 75% of patients with this deformity have neurofibromatosis type I [7].

Up to 15% of the patients with anterolateral bowing have fibrous dysplasia [5].

The clinical presentation varies considerably, from simple bowing to various extensive bone deformities.

Normally pseudarthosis of the tíbia is unilateral, located at the junction of the middle and distal thirds of the tibial segment with no predominance for sex or side.

Although there is consensus regarding the need for surgical intervention for established pseudoarthrosis, treatment of the anterolateral bowing is controversial. Many authors consider osteotomy of the bowed non-broken tibia a contraindication. However, leaving the patient with a deformed, bowed leg is intolerable and is associated with deformity progression and increased risk of fracture.

The standard treatment of bowing is bracing, which is somewhat successful in fracture prevention but not in stopping progression of the deformity. Many authors try to avoid bone osteotomy of the deformed bone to avoid the development of pseudoarthrosis. However, leaving the bone deformed is associated with difficulty in wearing an orthosis and progression of the development of secondary changes, which make the functional outcome of any future surgery poor [8].

Osteotomies of the lower extremity carry a variable risk of complications. These osteotomies may be opening wedge, closing wedge or a combination.

Osteotomies are often performed for Blount disease, achondroplasia, hypophosphatemic rickets and osteogenesis imperfect [9]. However, it could be possible seen the recurrence of bowing, and asymmetric growth plate closure [10].

Treatment of anterolateral bowing of the tibia is still challenging in pediatric orthopedics because of bone union difficulties, persistant angulation, joint stiffness and sometimes severe limb length discrepancy sequellae. Numerous treatments based on biological and/or mechanical concepts, surgical or not, have been reported with variable success rates [6].

## CASE REPORT

**Figure 1:**
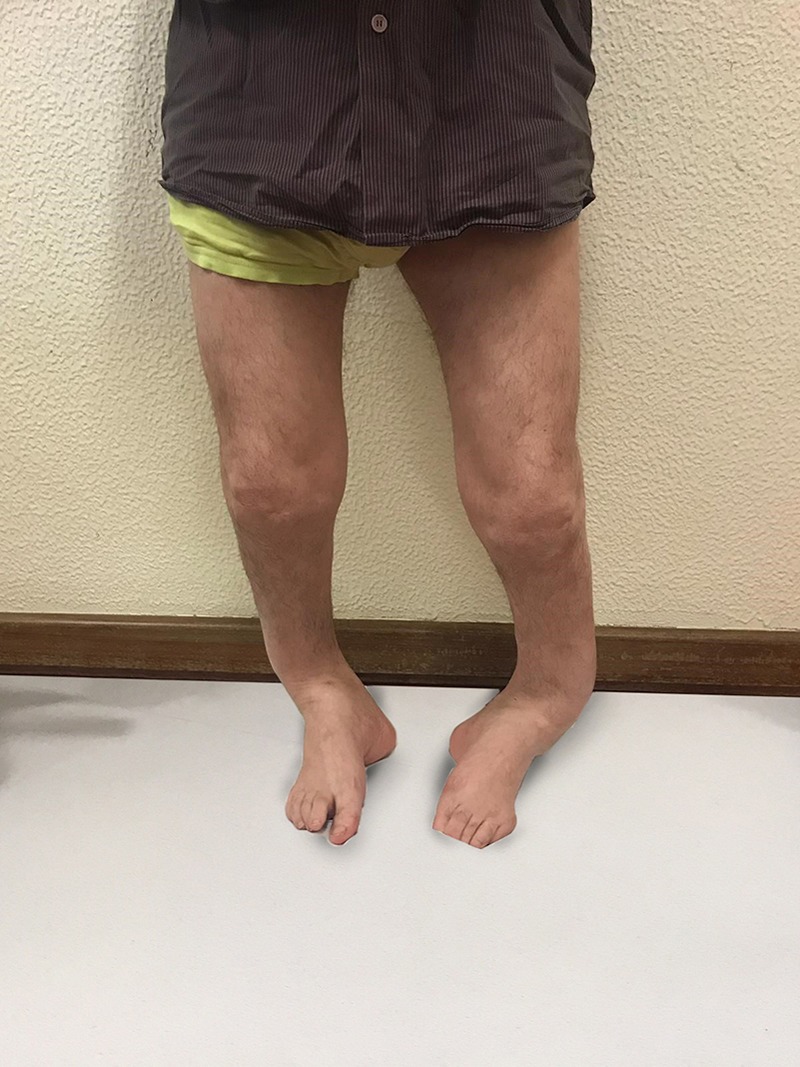
Lower limbs of his father.

A 6-year-old boy was referred to our department because of short stature and bilateral and symmetrical angular deformity of the lower limbs. Born at term by spontaneous vaginal delivery. His father has similar gait and limb deformity (Fig. 1). No other family history of relief.

On clinical examination, the patient was a short stature due to the disproportionate length of the legs. The disturbed gait was produced by the distal bowing of the tibia and he had callosity at the lateral border of the foot. The patient had no café-au-lait spots and normal cognitive achievement.

The children underwent a series of investigations, including complete blood cell counts, urine biochemistry, alkaline phosphatase and renal function parameters and tests, aimed at investigating calcium, phosphorus and vitamin D metabolism. However, all tests were normal. Hormonal investigations included thyroid hormones, adrenocorticotropic hormone and growth hormone which all were negative as well. The genetic study did not reveal any chromosomal anomaly.

**Figure 2:**
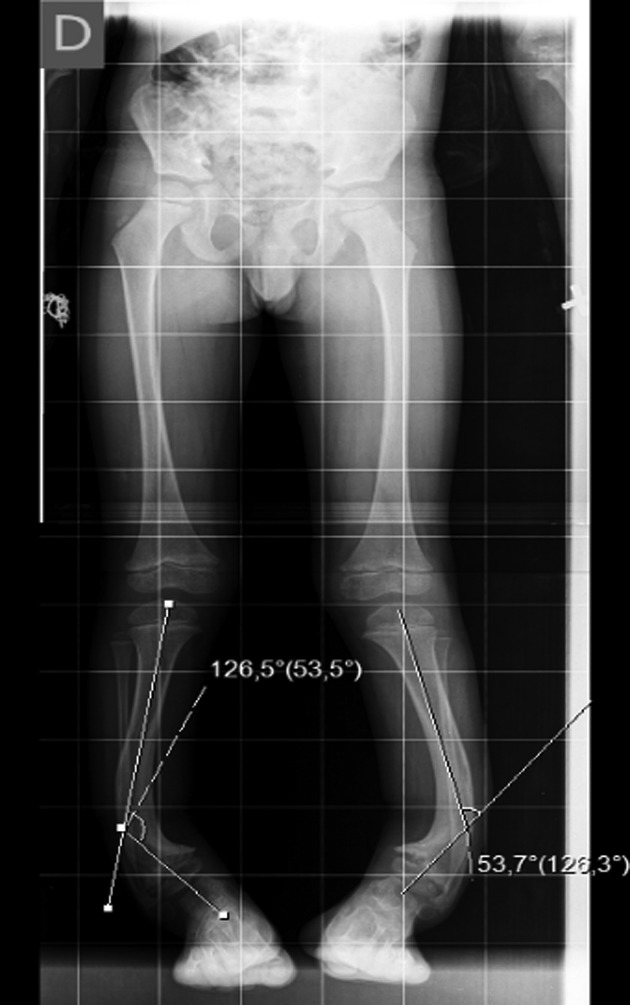
Preoperative Rx.

The X-ray showed a bowing of distal tibia, bilaterally, producing a varus deformity of 53 degrees (Fig. 2).

**Figure 3:**
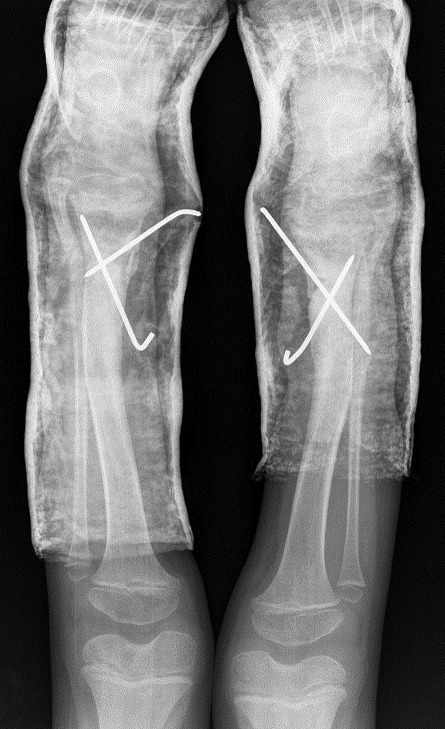
Posoperative Rx.

**Figure 4:**
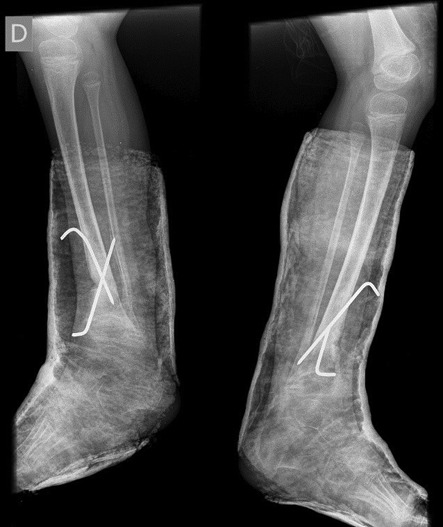
Posoperative Rx.

Surgical correction was scheduled. It was performed an opening wedge tibia osteotomy and filled the remaining gap with structural fibular graft. It was fixed with two crossed K-wires and cast immobilization. Both surgeries were performed at the same operative time (Figs 3 and 4).

The surgery was uneventful, after 6 weeks there was possible to observe healing of the osteotomy site so the cast and K-wires were removed.

**Figure 5:**
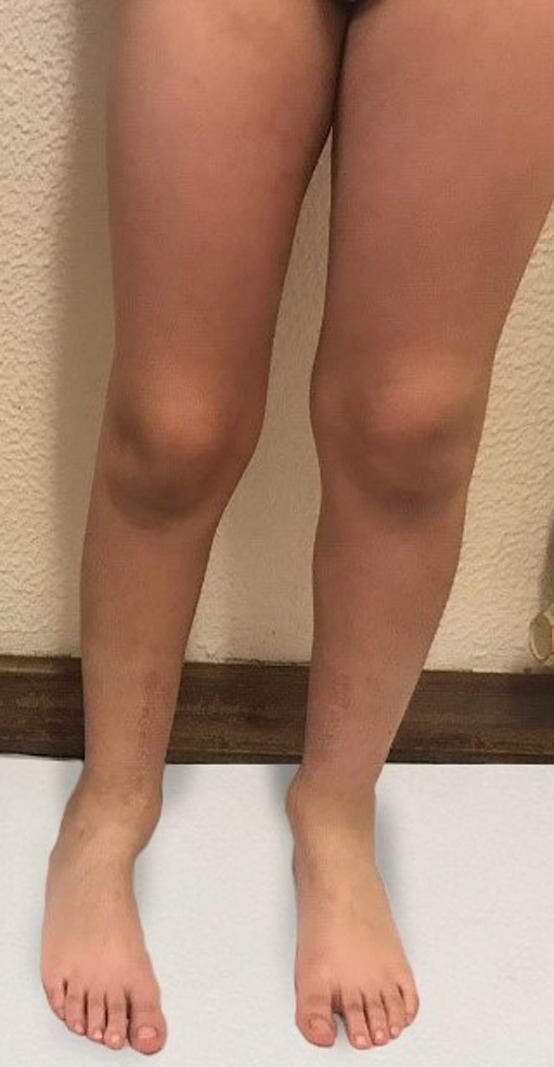
One year after the surgery.

**Figure 6:**
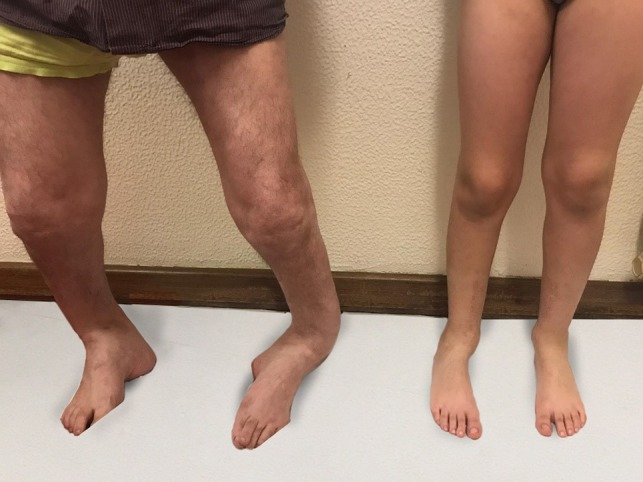
One year after the surgery - the comparison between the father and the child.

**Figure 7:**
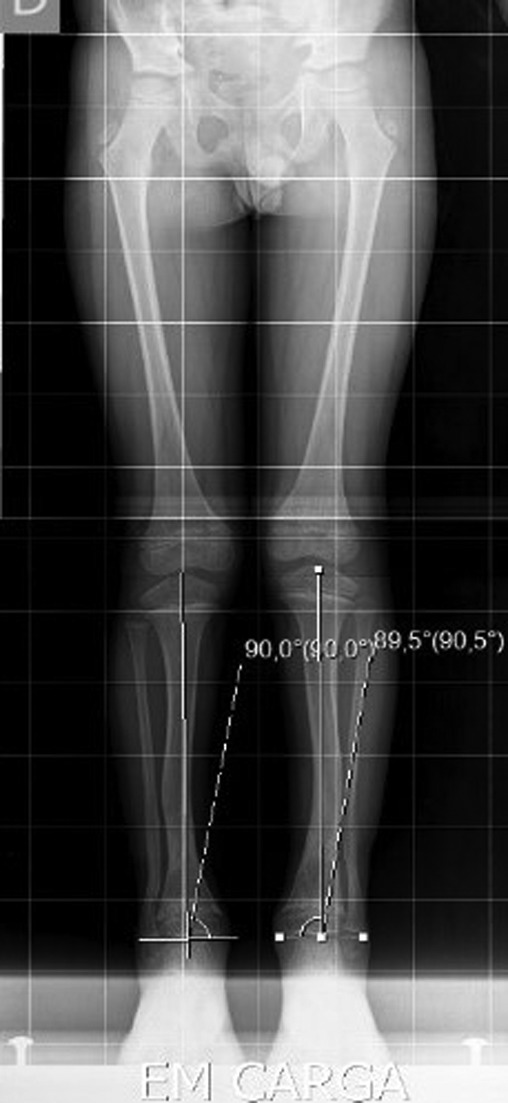
The Rx 1 year after the surgery.

After 1-year of follow-up, the patient presented with normal gait and no callosity (Figs 5 and 6). The long-leg standing X-rays demonstrated a correct alignment of the tibia in the anatomical and mechanical axis (Fig. 7).

## CONCLUSION

Bowing of the lower extremities is present in a wide variety of pathologic conditions. Recognition of these conditions is important for differentiating those that will resolve spontaneously from those that require surgery or other treatment.

Surgical treatment of bowing depends on the age of the patient and the cause and stage of the condition [10].

Although the best treatment for the anterolateral bowing remains controversial, there is a consensus on a certain number of points in the literature. Whatever the technique used [6], the goal of treatment is restoration of satisfactory mechanical alignment [10] and the realignment of the tibial segment and stable internal fixation are essential for union [6].

We report a case of non-dysplastic bilateral congenital anterolateral bowing of the tibia which corresponds to a type I of Crawford classification.

Given the significant disturbance of the gait, it was decided to correct it before skeletal maturity, with a satisfactory morphological and functional result.

Despite being the simplest form of congenital anterolateral bowing of the tibia, we describe an exuberant deformity with significant functional and esthetic impairment.

The technique used was simple and fast to perform, with no added risks of morbidity for the patient.
